# The complete mitogenome of the Thai soldier crab *Mictyris thailandensis* Davie, Wisespongpand & Shih, 2013 (Crustacea: Decapoda: Mictyridae)

**DOI:** 10.1080/23802359.2021.2024770

**Published:** 2022-01-27

**Authors:** Chutima Sonthirod, Sonicha U-thoomporn, Chaiwat Naktang, Wirulda Pootakham, Sithichoke Tangphatsornruang, Rueangrit Promdam, Aekkaraj Nualla-ong, Supattra Pongparadon

**Affiliations:** a National Omics Center, National Science and Technology Development Agency, Khlong Luang, Thailand; b Princess Maha Chakri Sirindhorn Natural History Museum, Faculty of Science, Prince of Songkla University, Songkhla, Thailand; c Division of Biological Science, Faculty of Science, Prince of Songkla University, Songkhla, Thailand; d Center for Genomics and Bioinformatics Research, Faculty of Science, Prince of Songkla University, Songkhla, Thailand; e Excellence Center for Biodiversity of Peninsula Thailand, Faculty of Science, Prince of Songkla University, Songkhla, Thailand

**Keywords:** *Mictyris thailandensis*, soldier crab, mitochondrial genome, phylogenetic analysis

## Abstract

*Mictyris thailandensis* has been described recently in the family Mictyridae which is found only in the Andaman Sea, west coast of Thailand. In this study, we performed shotgun genome sequencing of a male *M. thailandensis* using a paired-end (150 bp) sequencing chemistry on MGISEQ-2000RS and report the complete mitochondrial genome of *M. thailandensis* (15,557 bp). A total of 37 genes have been annotated: 13 protein-coding genes (PCGs), 22 transfer RNA genes (tRNAs), two ribosomal RNA genes (rRNAs), and a control region. Comparative phylogenetic analysis with 29 crustaceans based on 13 conserved genes demonstrated that *M. thailandensis* is closely related to other soldier crabs in the family Mictyridae. The mitogenome of *M. thailandensis* presented here provides useful genetic information to help understand the evolutionary relationships among the Mictyridae family members.

Soldier crabs of the genus *Mictyris* are inhabitants of a tidal flat in the estuary of the Indo-west Pacific region (Unno and Semeniuk 2016), include eight recognized species. *Mictyris thailandensis* Davie, Wisespongpand & Shih, 2013, is found only in the Andaman Sea, west coast of Thailand from Ranong to Satun Provinces, and has been described recently as a new species (Davie et al. 2013; Unno and Semeniuk 2016; WoRMS 2021). The morphology of this species can be recognized by the yellow-fawn walking legs, whitish chelipeds and noticeably broad upper palm. Its behaviors have been found to march in army, like other soldier crabs, and feed while walking. Moreover, the hummock-structure mounds created from subsurface feeding behavior is also found (Davie et al. 2013). *M*. *thailanden*sis and its congeners play a significant role in retaining healthy tidal flats where they live (Webb and Eyre 2004; Davie et al. 2013). To date, only one species from the genus *Mictyris* (*Mictyris longicarpus*) has the mitogenome available (Tan et al. 2014). The availability of the *M*. *thailanden*sis mitochondrial genome sequence will be valuable for future phylogenetic studies of this family. Here, the complete mitochondrial genome of *M*. *thailanden*sis has been assembled, annotated, and its phylogenetic relationships have been described.

In this study, five individuals of *M. thailandensis* were collected from Tambon Laem Son, Amphoe La-ngu, Satun Province, Thailand (6°55′00″ N, 99°41′00″ E). Sampling in this study was permitted by the Department of National Parks, Wildlife and Plant Conservation (project number P-17-51470). Collection and handling of the samples followed the guidelines of the Institutional Animal Care and Use Committee, Prince of Songkla University. The specimens were stored at the reference collection of Princess Maha Chakri Sirindhorn Natural History Museum, Prince of Songkla University, Thailand (psu.museum@gmail.com, Rueangrit Promdam, rueangrit.p@psu.ac.th) with the voucher number PSUZC-CRU-0086. The leg muscle tissue samples were collected from a male crab, snap-frozen in liquid nitrogen and stored at −80 °C until further use. Muscle tissue was homogenized in liquid nitrogen for DNA extraction using QIAamp Tissue Kit (Qiagen, Hilden, Germany). Approximately, 300 ng of genomic DNA were used for a library construction following the protocol in the MGIEazy FS Library Prep Kit (MGI Tech, Shenzhen, China). Paired-end (150 bp) sequencing was performed on the MGISEQ-2000RS according to the manufacturer’s instructions to obtain the shotgun genome sequences.

A total of 8.7 Gb cleaned read were obtained and assembled by MitoZ 2.4 (Meng et al. 2019). The complete *M. thailandensis* mitogenome, 15,557 bp in length (GenBank accession number MW697086), was annotated using the MITOS web server (Bernt et al. 2013). The genome contained 13 protein-coding genes (PCGs), 22 transfer RNA genes (tRNAs), two ribosomal RNA genes (rRNAs), and a control region. The overall nucleotide composition was 32.80% A (5103 nt), 11.27% G (1754 nt), 37.32% T (5806 nt), and 18.61% C (2894 nt). PCGs and RNA genes were confirmed using the Basic Local Alignment Search Tool (BLAST) (Altschul et al.^1990^). All of the PCGs used the canonical ATN initiation codons (eight with ATG, four with ATA, and one with ATT) and the typical stop codons (TAA or TAG) except for three genes (*COIII*, *ND5*, and *CYTB*), which showed incomplete stop codons (T––). The tRNA-coding genes ranged from 62 to 71 nucleotides, and the control region, located between rrnS and tRNA^Ile^, was 645 bp long with a significant AT bias (76.97%).

Thirteen PCGs of *M. thailandensis* and 29 crustaceans species were concatenated to perform multiple alignments by MAFFT (Katoh et al. 2019). Using the Neighbor-Joining algorithm with 1000 bootstrap replications, the phylogenetic tree was reconstructed by MEGA X 10.1 (Kumar et al. 2018) (Figure 1). *Drosophila melanogaster* was selected as an outgroup. Phylogenetic analysis showed that *M. thailandensis* was closely related to other soldier crab species in the family Mictyridae. The mitogenome of *M. thailandensis* presented here provides useful genetic information to help understand the evolutionary relationships among the Mictyridae family members.

**Figure 1.**
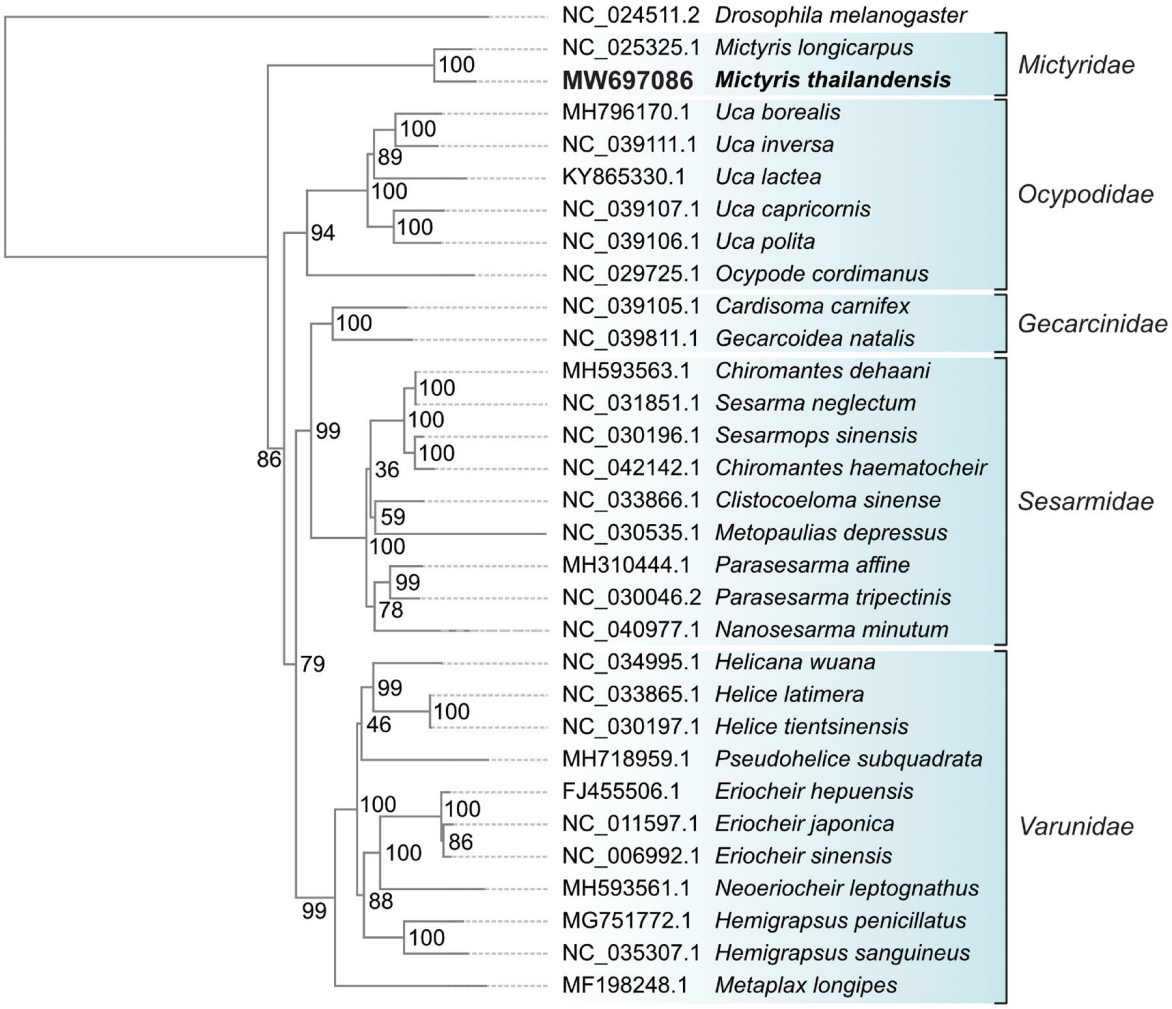
A neighbor-joining tree based on the 13 PCGs of *M. thailandensis* and 29 crustacean species. The bootstrap values are listed on each node, and the vertical line on the right side indicated a specific family in the class Crustacean.

## Authors contributions

SP, WP, and ST designed research study and obtained the funding. SU, RP, and AN performed laboratory work (sample collection, DNA extraction, library construction, and sequencing). CS and CN performed bioinformatics analyses. CS wrote and revised the manuscript, and all authors reviewed it.

## Data Availability

The data that support the findings of this study are openly available in GenBank of NCBI at https://www.ncbi.nlm.nih.gov/, accession number: MW697086.1. The associated BioProject, SRA, and Bio-Sample numbers are: PRJNA731290, SRR14597275, and SAMN19276314, respectively.

## References

[CIT0001] Altschul S, Gish W, Miller W, Myers E, Lipman D. 1990. Basic local alignment search tool. J Mol Biol. 215(3):403–410. 2231712 10.1016/S0022-2836(05)80360-2

[CIT0002] Bernt M, Donath A, Jühling F, Externbrink F, Florentz C, Fritzsch G, Pütz J, Middendorf M, Stadler PF. 2013. MITOS: improved de novo metazoan mitochondrial genome annotation. Mol Phylogenet Evol. 69(2):313–319. 22982435 10.1016/j.ympev.2012.08.023

[CIT0003] Davie PJ, Wisespongpand P, Shih H. 2013. A new species of *Mictyris* Latreille, 1806 (Crustacea: Decapoda: Brachyura: Mictyridae) from the Andaman coast of Thailand, with notes on its ecology and behavior. Zootaxa. 3686(1):65–76. 26473206 10.11646/zootaxa.3686.1.3

[CIT0004] Katoh K, Rozewicki J, Yamada KD. 2019. MAFFT online service: multiple sequence alignment, interactive sequence choice and visualization. Brief Bioinform. 20(4):1160–1166. 28968734 10.1093/bib/bbx108 PMC6781576

[CIT0005] Kumar S, Stecher G, Li M, Knyaz C, Tamura K. 2018. MEGA X: molecular evolutionary genetics analysis across computing platforms. Mol Biol Evol. 35(6):1547–1549. 29722887 10.1093/molbev/msy096 PMC5967553

[CIT0006] Meng G, Li Y, Yang C, Liu S. 2019. MitoZ: a toolkit for ANIMAL mitochondrial genome assembly, annotation and visualization. Nucleic Acids Res. 47(11):e63. 30864657 10.1093/nar/gkz173 PMC6582343

[CIT0007] Tan MH, Gan HM, Lee YP, Austin CM. 2014. The complete mitogenome of the soldier crab *Mictyris longicarpu*s (LATREILLE, 1806) (Crustacea: Decapoda: Mictyridae). Mitochondrial DNA. 27:1–2. 25423510 10.3109/19401736.2014.982585

[CIT0008] Unno J, Semeniuk V. 2016. Soldier crabs (Mictyridae). In: Kennish MJ, editors. Encyclopedia of Estuaries. Encyclopedia of Earth Sciences Series. Dordrecht: Springer.

[CIT0009] Webb AP, Eyre BD. 2004. The effect of natural populations of the burrowing and grazing soldier crab (*Mictyris longicarpus*) on sediment irrigation, benthic metabolism and nitrogen fluxes. J Exp Mar Biol Ecol. 309(1):1–19.

[CIT0010] WoRMS. 2021. *Mictyris* Latreille, 1806; [accessed 2021 Mar 26]. https://www.marinespecies.org/aphia.php?p=taxdetails&id=439492

